# The accuracy of digital templating in cementless total hip arthroplasty in dysplastic hips

**DOI:** 10.1186/s12891-021-04793-6

**Published:** 2021-11-10

**Authors:** Emelie Kristoffersson, Volker Otten, Sead Crnalic

**Affiliations:** Department of Surgical and Perioperative Sciences (Orthopaedics), Umeå University, 90185 Umeå, Sweden

**Keywords:** Digital templating, Dysplasia, Cementless total hip arthroplasty, Accuracy

## Abstract

**Background:**

Total hip arthroplasty (THA) for developmental dysplasia of the hip (DDH) is a complex procedure due to associated anatomical abnormalities. We studied the extent to which preoperative digital templating is reliable when performing cementless THA in patients with DDH.

**Methods:**

We templated and compared the pre- and postoperative sizes of the acetabular and femoral components and the center of rotation (COR), and analysed the postoperative cup coverage, leg length discrepancy (LLD), and stem alignment in 50 patients (56 hips) with DDH treated with THA.

**Results:**

The implant size exactly matched the template size in 42.9% of cases for the acetabular component and in 38.2% of cases for the femoral component, whereas the templated ±1 size was used in 80.4 and 81.8% of cases for the acetabular and femoral components, respectively. There were no statistically significant differences between templated and used component sizes among different DDH severity levels (acetabular cup: *p* = 0.30 under the Crowe classification and *p* = 0.94 under the Hartofilakidis classification; femoral stem: *p* = 0.98 and *p* = 0.74, respectively). There were no statistically significant differences between the planned and postoperative COR (*p* = 0.14 horizontally and *p* = 0.52 vertically). The median postoperative LLD was 7 (range 0–37) mm.

**Conclusion:**

Digital preoperative templating is reliable in the planning of cementless THA in patients with DDH.

## Background

Total hip arthroplasty (THA) for developmental dysplasia of the hip (DDH) is a complex procedure due to the associated anatomical abnormalities, such as anteversion of the proximal femur, a narrow femoral canal and the modified shape of the acetabulum [1]. Furthermore, the leg length discrepancy (LLD) in patients with DDH is often greater than in patients with primary osteoarthritis due to subluxation of the femoral head and the creation of a false acetabulum above the anatomical acetabulum. Therefore, restoration of optimal biomechanical conditions, including the optimal placement of the prosthetic components and avoidance of unwanted LLD, are important aspects to consider when planning and performing THA in these patients [2, 3].

Preoperative templating is important in achieving a successful outcome in THA, but there are few reports on the accuracy and reliability of digital templating for THA in patients with DDH [3–5]. Gamble et al. analysed the accuracy of digital preoperative templating compared to conventional preoperative templating for primary cementless THA but excluded patients with DDH [5]. Unnanuntana et al. evaluated the accuracy of preoperative acetate templating in terms of determining the final implant size, position and LLD in cementless THA due to both primary and secondary osteoarthritis [4]. Their study revealed a lower accuracy for preoperative templating of the acetabular component (42.2%) than for the femoral component (68.8%), which they attributed to the fact that a large portion of the cases in the study (62.4%) suffered from DDH. Zhao et al. assessed the utility of digital preoperative templating in patients with Crowe type II and III dysplastic hips compared with a control group with other primary diagnoses and found that the predictability for the cup size was significantly lower in the dysplastic hip group, but there was no significant difference in predicting the femoral component size [3]. Thus, there is still a need to optimize the planning of THA in patients with DDH.

The aim of this study was to evaluate how well the implanted component sizes and postoperative implant position corresponded to the preoperative digital templating for cementless THA in patients with DDH.

## Methods

We reviewed the preoperative and 1–3 days postoperative radiographs of 50 consecutive patients (27 women and 23 men), 56 hips, who underwent cementless THA due to osteoarthrosis secondary to DDH between February 2008 and September 2015 at the Department of Orthopaedics, Umeå University Hospital.

The center-edge (CE) angle was measured as described by Wiberg [6]. Patients with a CE angle less than 25° were included in the study.

The type and severity of DDH were classified according to the Crowe and Hartofilakidis classifications [7, 8]. The Crowe classification system is based on the amount of subluxation of the femoral head: type I: < 50% subluxation, type II: 50–74% subluxation, type III: 75–99% subluxation and type IV: completely dislocated. The degree of subluxation was calculated as L = d/(h/5), where L is the degree of subluxation, d is the perpendicular distance between the interteardrop line and the head-neck junction of the dysplastic femoral head and h is the height of the pelvis measured as the vertical distance from the highest point of the iliac crest perpendicular to a line drawn connecting the ischial tuberosities [7].

Eight patients lacked anteroposterior (AP) radiographs showing the height of the pelvis. Of these, 6 patients had unilateral dysplasia, and the degree of subluxation was calculated using the vertical height of the femoral head of the “normal” hip, which Crowe et al. have determined to be one-fifth of the pelvic height [7]. The remaining 2 patients (4 hips) without AP radiographs showing the pelvic height had bilateral dysplasia; therefore, classification according to Crowe was not possible, and they were excluded from the Crowe classification groups.

The classification system proposed by Hartofilakidis is based on the description of anatomical abnormalities [8]. Type A, also called dysplasia, refers to a hip with the femoral head within the true acetabulum despite some subluxation and inadequate depth of the acetabulum. In both type B (low dislocation) and type C (high dislocation), the femoral head has migrated superiorly in different extensions with complete uncovering by the true acetabulum in type C.

### Preoperative templating

Preoperative radiological templating was performed using the Mdesk™ system (RSA Biomedical, Umeå, Sweden). Standard radiographs of the hip included AP and cross-table lateral views. The AP pelvis radiograph was made with the patient supine, and both legs internally rotated approximately 15° degrees. A marker ball with a diameter of 30 mm, placed in the same coronal plane as the hip when taking the preoperative AP radiographs, was used for image calibration before performing digital templating. Preoperative templating was performed by the same surgeon who later performed the THA.

**Fig. 1:**
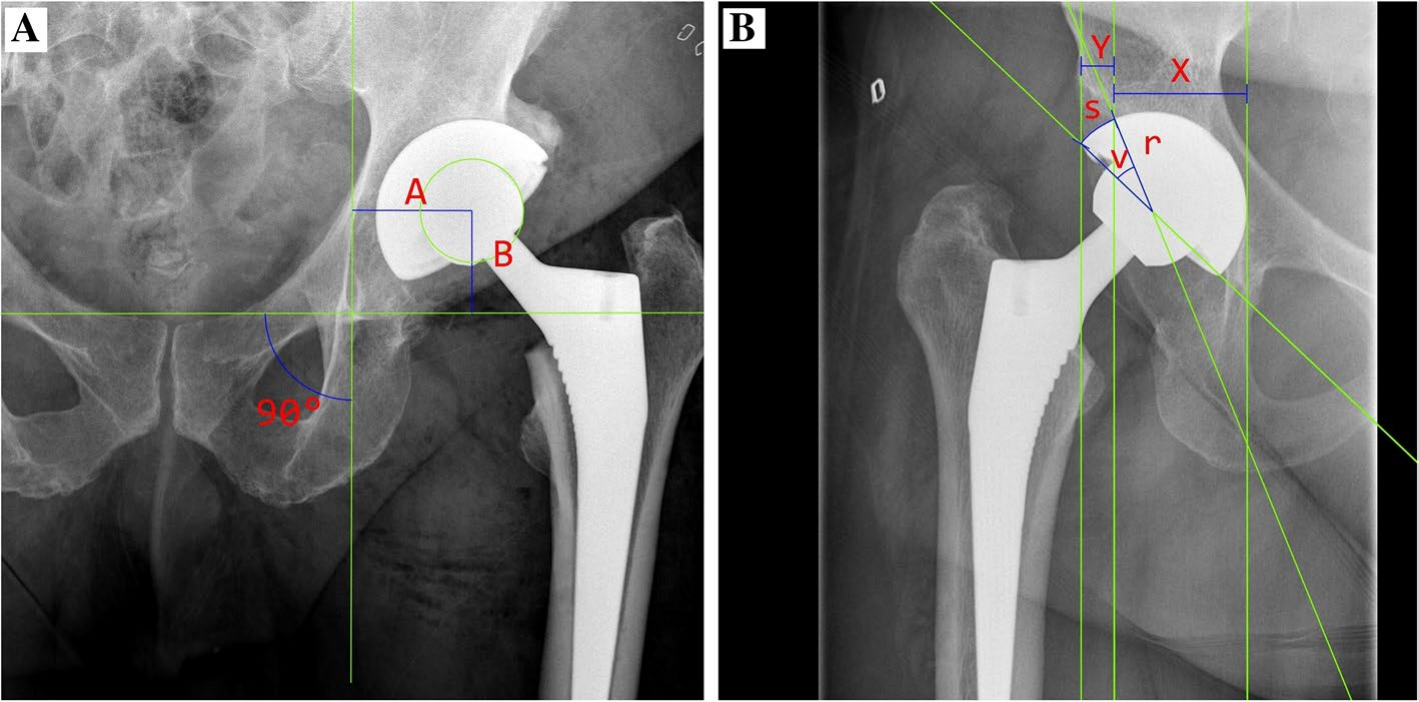
**A** Postoperative anteroposterior radiograph of a left Crowe type II, Hartofilakidis type B hip with measurement of location of hip centre with **A** the horizontal location of hip centre and **B** the vertical height of the hip centre. **B** Postoperative anteroposterior radiograph showing the assessment of projected proportion of horizontal uncovering above the acetabular component, calculated as (horizontal distance of the uncovered portion (Y) / horizontal distance between the medial and lateral edge of the implant (X)) × 100% [11]. The arc length (s) of the uncovered portion above the implant was calculated using the formula: s = (v x *r* x π) / 180, where v denotes the central angle of the uncovered portion above the implant and *r* the radius of the component [9]

The following measurements were made on the preoperative radiographs:

1. The templated horizontal and vertical positions of the center of rotation (COR) on the AP radiograph were determined (Fig. 1A) [9].
2. The preoperative LLD was determined on the AP radiograph as the difference between the right and left hip of the perpendicular distance from the interischial line to the proximal junction of the lesser trochanter.

The following measurements were made on the postoperative radiographs that were calibrated using the known size of the prosthetic femoral head:

1. The postoperative vertical and horizontal positioning of the COR was measured in the same way as the preoperative measurement, and the difference compared to the preoperative planning was calculated [9].
2. The postoperative inclination angle was defined as the abduction angle between the horizontal interteardrop line and a line drawn though the opening of the acetabular cup on the AP radiograph.
3. The version angle of the cup was determined from the cross-table lateral view radiograph as described by Yao et al. [10]
4. The coverage of the cup was determined as described in Fig. 1B [9, 11].
5. The femoral stem alignment was measured on the AP radiograph as the angle between the longitudinal axis of the proximal femur and the longitudinal axis of the femoral component.
6. The postoperative LLD was measured on the AP radiograph as the perpendicular distance from the interischial line to the proximal junction of the lesser trochanter of both sides.

The final prosthetic component sizes were obtained from operative notes.

### Surgical procedure

All the operations were performed using a posterior approach by five different surgeons. Nine different acetabular components were used: 21 Pinnacle (DePuy, Warsaw, Ind), 14 Reflection (Smith&Nephew, Richards Inc., Memphis, TN), 8 Avantage (Biomet), 4 Trilogy (Zimmer, Warsaw, IN), 4 Continuum (Zimmer, Warsaw, IN), 2 articular surface replacement (ASR) (DePuy, Warsaw, IN), 1 Trident peripheral self-locking (PSL) (Stryker, Rutherford, NJ), 1 Bantam (DePuy, Warsaw, IN) and 1 Trabecular metal revision shell (Zimmer, Warsaw, IN). Five different femoral components were used: 47 Corail (DePuy, Warsaw, IN), 4 Wagner cone (Zimmer, Warsaw, IN), 3 Synergy (Smith&Nephew, Richards Inc., Memphis, TN), 1 CLS (Zimmer, Warsaw, IN) and 1 custom-made stem (Unique, SCP, Trondheim).

In seven hips, autotransplantation of bone from the femoral head was used to restore the acetabulum and optimize coverage of the acetabular cup. Complete coverage was achieved in all 7 of these cases. Two hips (both Crowe type IV) underwent femoral osteotomy and were therefore excluded when analysing postoperative LLD.

### Statistics

Descriptive statistics of continuous variables were expressed as median (range), while categorical data were expressed as numbers and percentages. The proportions were compared with the Fisher’s exact tests. The Wilcoxon matched-pairs signed rank test was used to determine the difference between the preoperative and postoperative position of the COR. A *p*-value of ≤0.05 was considered statistically significant. Statistical analyses were performed using GraphPad Prism 6.0 software for Windows (GraphPad Inc., San Diego, CA).

## Results

Table 1 summarises the clinical characteristics of the patients.

**Table 1:** Patient characteristics^a^

	Crowe classification (***n*** = 52 hips^**b**^)			Hartofilakidis classification (***n*** = 56hips)			Total (***n*** = 56)
	Type I (***n*** = 39)	Type II (***n*** = 8)	Types III, IV (***n*** = 5)	Type A (***n*** = 30)	Type B (***n*** = 22)	Type C (***n*** = 4)	
**Sex W/M**	20/19	5/3	5/0	15/15	13/9	4/0	32/24
**Age (range-yr)**	23–68	32–66	28–68	26–68	23–67	28–68	23–68
**BMI**	25.8 (17.4–44.8)	24.8 (22.3–39.6)	36.1 (21.6–39.4)	26 (17.4–44.8)	26.8 (22.3–43.8)	23.4 (21.6–38.2)	26 (17.4–44.8)
**Operated side** **- left/right**	17/22	2/6	3/2	15/15	7/15	2/2	24/32
**ASA**^**c**^**class 1**	12	1	2	6	7	2	15
**ASA class 2**	24	4	1	22	9	2	33
**ASA class 3**	1	3	2	0	6	0	6
**%subluxation**^**d**^	13.5 (0–48.2)	62.7 (50.8–70.7)	120.2 (76.7–168.4)	–	–	–	22.7 (0–168.4)

^a^Data are presented as the median with the range in parentheses or as the number of hips

^b^Two patients (four hips) were excluded due to the absence of radiographs

^c^Data was missing for two patients

^d^Degree of proximal subluxation of the femoral head as described by Crowe et al. [7]

For the implanted acetabular component, the exact size was templated in 42.9% of cases, with an increase to 80.4% when using a size within one size (above or below) of the templated size (Table 2). For the implanted femoral stem, the exact size was templated in 38.2% of cases, and in 81.8% of cases, the used size was within one size (above or below) of the templated size. When the implanted acetabular component size did not match the templated component size, 19 acetabular components (59.4%) were smaller than what was templated. There were no statistically significant differences in the reliability of templating among the Crowe classification groups or the Hartofilakidis classification groups for either the acetabular component (*p* = 0.30 and *p* = 0.94, respectively) or for the femoral stem (*p* = 0.98 and *p* = 0.74, respectively).

**Table 2:** Accuracy (%) of the preoperative templating of the prosthetic component sizes

	Crowe classification (***n*** = 52 hips^**a**^)			Hartofilakidis classification (***n*** = 56 hips)			Total accuracy
	Type I (***n*** = 39)	Type II (***n*** = 8)	Types III, IV (***n*** = 5)	Type A (***n*** = 30)	Type B (***n*** = 22)	Type C (***n*** = 4)	(***n*** = 56)
**Accurate size of the cup**	41.0	62.5	20	43.3	40.9	50	42.9
**Size of the cup within ± 1 size**	76.9	100	80	83.3	77.3	75	80.4
**Accurate size of the stem**	36.0	37.5	50^b^	33.3	40.9	66.7^b^	38.2
**Size of the stem within ± 1 size**	79.5	87.5	100^b^	76.7	86.3	100^b^	81.8

^a^Two patients (4 hips) were excluded due to the absence of radiographs

^b^One patient received a custom-made stem and was excluded

For the templated COR, the median horizontal distance was 29 (18–90) mm and the median vertical distance was 20 (11–65) mm. On postoperative radiographs, the COR had a median distance of 29 (20–87) mm horizontally and 20 (8–63) mm vertically (*p* = 0.14 and *p* = 0.52, respectively) (Table 3). The majority of acetabular components (80.4%) were placed less than 25 mm superior to the interteardrop line. Only 1 acetabular cup was placed more than 35 mm superior to the interteardrop line. For the horizontal placement of the COR, 14.5% of acetabular components were placed at or within a 25 mm horizontal distance from the teardrop.

**Table 3:** Predicted and postoperative locations of the hip center^a^

	Crowe classification (***n*** = 52 hips^**b**^)			Hartofilakidis classification (***n*** = 56 hips)			Total (***n*** = 56)
	Type I (***n*** = 39)	Type II (***n*** = 8)	Type III, IV (***n*** = 5)	Type A (***n*** = 30)	Type B (***n*** = 22)	Type C (***n*** = 4)	
**Horizontal location of the hip center (mm)**							
**Predicted**	29 (23–39)	26 (18–90)	26 (18–28)	29.5 (23–39)	28 (18–33)	23 (18–90)	29 (18–90)
**Postoperative**	29 (24–38)	31 (21–87)	26 (20–33)	29.5 (24–38)	29.5 (21–36)	24 (20–87)	29 (20–87)
**Vertical location of the hip center (mm)**							
**Predicted**	20 (13–34)	21 (11–65)	19 (12–26)	20 (13–34)	21 (11–31)	15 (12–65)	20 (11–65)
**Postoperative**	20 (11–37)	27 (22–63)	29 (8–34)	19.5 (11–37)	22 (16–34)	12 (8–63)	20 (8–63)

^a^Data are presented as the median with the range in parentheses

^b^Two patients (4 hips) were excluded due to the absence of radiographs

Complete coverage was observed in 17 acetabular components (30.4%), of which seven hips required autogenous bone grafts (Table 4). Only 4 acetabular components had less than 75% horizontal coverage. The median arc length of the uncovered portion of the acetabular component was 6.3 (0–16.2) mm.

**Table 4:** Postoperative orientation and horizontal coverage of the acetabular component

	Crowe classification (***n*** = 52 hips^**a**^)			Hartofilakidis classification (***n*** = 56 hips)			Total (***n*** = 56)
	Type I (***n*** = 39)	Type II (***n*** = 8)	Types III, IV (***n*** = 5)	Type A (***n*** = 30)	Type B (***n*** = 22)	Type C (***n*** = 4)	
**Inclination**^**b**^	43 (22–63)	40.5 (36–54)	47 (39–55)	46.5 (22–63)	40 (22–54)	53.5 (39–59)	43 (22–63)
**Anteversion**^**b**^	18.5 (2–40) (*n* = 34)	21.5 (14–34) (*n* = 6)	24 (8–38) (*n* = 5)	19.5 (2–40) (*n* = 28)	19 (6–27) (*n* = 17)	29 (10–38) (*n* = 4)	19 (2–40) (*n* = 49)
**Retroversion**^**b**^	3 (2–7) (*n* = 5)	8 (5–11) (*n* = 2)	0	2.5 (2–3) (*n* = 2)	6 (2–11) (*n* = 5)	0	5 (2–11) (*n* = 7)
**Coverage %**^**c**^							
Complete 100	11	4	2	8	7	2	17
90–99	5	1	1	5	2	1	8
75–89	20	2	2	15	11	1	27
< 75	3	1	0	2	2	0	4

^a^Two patients (4 hips) were excluded due to the absence of radiographs

Data are presented as the ^b^median with the range in parentheses or as the ^c^number of hips

**Fig. 2:**
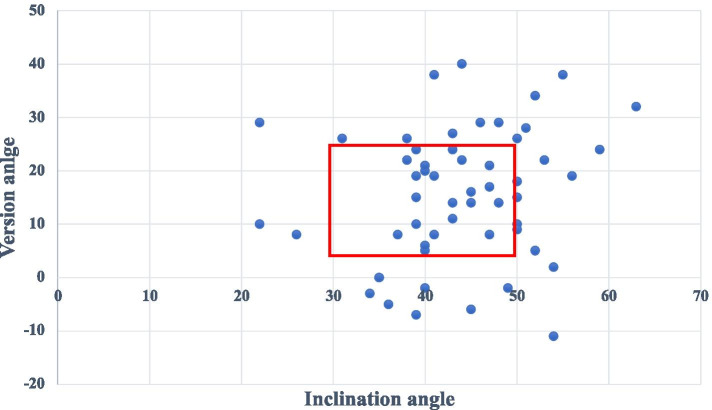
Correlation between radiographic inclination and anteversion angles of the acetabular component. The marked area represents the assumed “safe zone” described by Lewinnek et al. [12]

The median cup inclination angle was 43 (22–63)° (Table 4). Ten acetabular components had an inclination angle greater than 50°, and two cups had an inclination angle less than 30°. Forty-nine cups were anteverted at a median angle of 19 (2–40)°, and 7 cups were placed in retroversion at a median angle of 5 (2–11)°. Fifty percent of the acetabular cups were placed within the assumed “safe zone” described by Lewinnek et al. (Fig. 2) [12].

The median LLD was 16.3 (0–44) mm preoperatively and 7 (0–37) mm postoperatively. The postoperative LLD was not measurable on two hips due to femoral osteotomy (both Crowe type IV and Hartofilakidis type C).

Postoperatively, 13 femoral components were positioned in a neutral position, 28 femoral components were positioned in varus [median 2 (1–8)°] and 15 were positioned in valgus [median 1 (1–3)°].

**Fig. 3:**
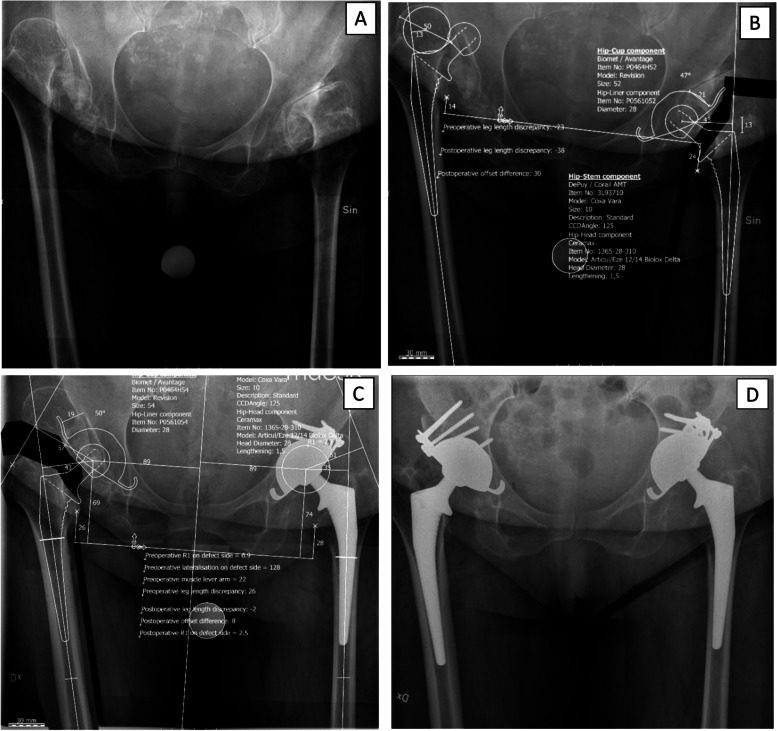
**A** Preoperative AP radiograph of patient with bilateral DDH: left hip Crowe type III and Hartofilakidis type B, right hip Crowe type IV and Hartofilakidis type C. **B** Preoperative templating of the left hip. **C** Preoperative templating of the right hip. **D** Postoperative AP radiograph after bilateral THA

Figure 3 shows digital templating for THA in one patient with bilateral hip dysplasia.

## Discussion

The goal of this study was to evaluate whether preoperative digital templating is reliable when planning cementless THA in patients with DDH. We found high accuracy for predicting the prosthetic component sizes and for placement of COR.

Our results show that the accuracy for templating exact component sizes is 42.9% for acetabular components and 38.2% for femoral components, but when templating within ±1 size, the accuracy was 80.4 and 81.8% for acetabular and femoral components, respectively. Eggli et al. and Gonzalez Della Valle et al. found a higher accuracy than in the present study, but they studied mainly cemented THA [13, 14]. When templating cementless components, the accuracy of the predicted components’ sizes is generally lower than for cemented components, which may have originated from the press-fit technique for cementless implants [4]. Carter et al. achieved a 50% accuracy for predicted cementless femoral components with acetate templating [15]. With the introduction of digital templating, studies comparing the precision between digital and acetate preoperative templating have concluded that digital templating is a reliable tool and that the accuracy for templating increases over time as the surgeons become more familiar with the planning programs [5, 16–19].

Few studies have been carried out on the reliability of preoperative templating in patients with DDH. Unnanuntana et al. studied a mixed patient group consistent with 62.4% patients with DDH with the aim of evaluating the reliability of manual preoperative templating and whether the status of the contralateral hip affects the accuracy of the templated component size [4]. They found a higher but not statistically significant precision for templating the correct component size when the contralateral hip was not dysplastic. Zhao et al. studied the validity of digital preoperative templating for cementless THA in patients with DDH and found a precision of 48.8% for templating the acetabular component (within ±1 size) and a 73.2% precision for templating the femoral stem (within ±1 size) [3]. One reason for their lower precision compared to what we found could be that the most of our patients had less severe dysplasia. Zhao et al. also included a control group consistent with patients with other primary diagnoses in need of THA and found a significantly lower accuracy in predicting the acetabular component size in patients with DDH [3]. They concluded that the low accuracy of templating the acetabular component size might stem from the difficulty in predicting the vertical distance of the COR. In contrast, the results in our study show a good reliability when predicting the vertical distance of the COR, which can be another explanation for the higher precision when templating the acetabular component size in our study.

We did not find any significant differences in the accuracy between different classifications of DDH for size templating either for the acetabular component or the stem. However, our patients were unevenly spread across the Crowe classification groups, with 75% of patients in the Crowe type I group, making the analysis between DDH groups ambiguous.

We found a lower accuracy for templating the size of the femoral component than for templating the size of the acetabular component, which may derive from the abnormal anatomy of the femoral canal and difficulties in mapping the anatomy on regular two-dimensional radiographs [1]. Additionally, many patients with DDH suffer from reduced mobility, especially for rotation of the hip, which makes it harder to take optimal radiographs and might further add to the difficulties of radiographic visualization.

Several studies on long-term effects on the loosening rate and polyethylene annual wear on the acetabular component depending on horizonal and vertical placement of the COR have been carried out and agree that the optimal placement of the COR is less than 35 mm vertically from the interteardrop line and less than 25 mm laterally from the teardrop [11, 20–22]. Placement of the COR in a superolateral position has also been shown to affect the abductor muscles negatively by decreasing the lever arm and the force-generating capacity of the abductor muscles [23]. Some studies suggest a superior positioning of the COR if positioning in the true acetabulum is impossible, but priority should be given to recreate true anatomical measures [8, 22, 24].

In this study, the accuracies for predicting both the horizontal and vertical position of the COR were high, without significant differences between different dysplasia groups. The placement of the COR differs little from that in earlier studies, which found an average femoral head placement of 27.7–30.4 mm horizontally and 21.2–23.7 mm vertically [11, 20, 22]. The anatomical abnormalities that provide challenges for reconstruction differ according to different types and severities of DDH. The challenge in patients with Crowe type I and II and Hartofilakidis type A hips mainly lies in detecting and recreating the dysplastic, shallow anatomical acetabulum. In patients with Crowe type III and IV and Hartofilakidis type B and C hips, the subluxation of the femoral head leads to deficient acetabular bone stock and difficulties in acetabular component fixation. These difficulties might explain why the mean postoperative horizontal distance to the COR in our study is slightly greater for patients with Crowe type I and Hartofilakidis type A hips compared to hips with more severe preoperative subluxation.

The main limitation of our study is the retrospective approach causing the exclusion of patients lacking adequate radiographs. Most postoperative measurements were made on plain two-dimensional radiographs taken 1–3 days after surgery, which are rarely optimal. Last, our patient population was rather small; a majority of patients had less severe dysplasia making it hard to draw statistically significant conclusions and compare the accuracy between different DDH groups.

## Conclusion

Our study confirmed that digital preoperative templating could be followed in the majority of cases when performing THA in patients with DDH.

## Data Availability

The datasets used and/or analysed during the current study are available from the corresponding author on reasonable request.

## Abbreviations

THA: Total hip arthroplasty
DDH: Developmental dysplasia of the hip
LLD: Leg length discrepancy
CE angle: Center-edge angle
AP: Anteroposterior
COR: Center of rotation
